# Postoperative Residual Neuromuscular Paralysis at an Australian Tertiary Children's Hospital

**DOI:** 10.1155/2015/410248

**Published:** 2015-05-10

**Authors:** Thomas Ledowski, Brendan O'Dea, Luke Meyerkort, Mary Hegarty, Britta S. von Ungern-Sternberg

**Affiliations:** ^1^School of Medicine and Pharmacology, University of Western Australia, Perth 6009, WA, Australia; ^2^Department of Anaesthesia and Pain Medicine, Royal Perth Hospital, Perth 6000, WA, Australia; ^3^Department of Anaesthesia and Pain Management, Princess Margaret Hospital for Children, Perth 6008, Australia

## Abstract

*Purpose*. Residual neuromuscular blockade (RNMB) is known to be a significant but frequently overlooked complication after the use of neuromuscular blocking agents (NMBA). Aim of this prospective audit was to investigate the incidence and severity of RNMB at our Australian tertiary pediatric center. *Methods*. All children receiving NMBA during anesthesia were included over a 5-week period at the end of 2011 (Mondays to Fridays; 8 a.m.–6 p.m.). At the end of surgery, directly prior to tracheal extubation, the train-of-four (TOF) ratio was assessed quantitatively. Data related to patient postoperative outcome was collected in the postoperative acute care unit. *Results*. Data of 64 patients were analyzed. Neostigmine was given in 34 cases and sugammadex in 1 patient. The incidence of RNMB was 28.1% overall (without reversal: 19.4%; after neostigmine: 37.5%; n.s.). Severe RNMB (TOF ratio < 0.7) was found in 6.5% after both no reversal and neostigmine, respectively. Complications in the postoperative acute care unit were infrequent, with no differences between reversal and no reversal groups. *Conclusions*. In this audit, RNMB was frequently observed, particularly in cases where patients were reversed with neostigmine. These findings underline the well-known problems associated with the use of NMBA that are not fully reversed.

## 1. Introduction

In pediatric anesthesia, neuromuscular blocking agents (NMBA) are frequently used to facilitate tracheal intubation [1], positive pressure ventilation, and optimal surgical operating conditions [2]. Recently, some publications in adults [3–6] have found that deep neuromuscular blockade significantly improves intra-abdominal space and surgical working conditions throughout laparoscopic procedures. However, the use of NMBA exposes the patient to the well-described risk of residual neuromuscular blockade (RNMB) [7]. In adults, many studies have estimated the risk of RNMB to range anywhere from 3.5% to 83% [8]. In children, however, the available data is extremely sparse [2] with just one publication investigating the matter within the last decade [9]. The risks associated with RNMB are commonly underestimated by anesthesiologists, even though the pathophysiological consequences of residual paralysis may be severe [8]. The use of NMBA has been associated with an increased risk for respiratory adverse events in children undergoing anesthesia, particularly in the postoperative period [10], as well as with more direct impairments of lung function [11].

It was the aim of this prospective audit, therefore, to identify the rate and severity of RNMB at our tertiary pediatric center.

## 2. Methods

Ethics approval was granted for this project from the Princess Margaret Hospital Ethics Committee (457 QP) and recognized by the Human Research Ethics Office of the University of Western Australia (RA/4/1/5966). Written informed consent was waived since this audit was classified as a quality of care audit due to its purely observational character.

All children undergoing elective or urgent surgery under general anaesthesia requiring an endotracheal tube and receiving NMBA at Princess Margaret Hospital for Children in Perth, Western Australia, during the time period from 11/9/2011 to 12/16/2011 were targeted for this audit. Exclusions included patients operated on outside normal working hours (Monday to Friday 8 a.m. to 6 p.m.), emergency procedures, children who were not planned to be extubated in the operating theatre, and infants under 12 months who were too small for the available monitoring electrodes to be attached.

Data recorded included basic demographic information, as well as preexisting medical conditions and preoperative medications, data related to surgery and anesthesia (i.e., type of surgery, time of surgery and anesthesia, method of anesthesia, and NMBA used), and adverse events recorded in the postoperative acute care unit (PACU).

At the end of the surgical procedure and when the attending anesthesiologist deemed it safe to extubate the child, the train-of-four (TOF) ratio was assessed by an independent researcher using acceleromyometry (TOF Watch; Organon Teknika; Durham, North Carolina, USA) via a supramaximal stimulation of the ulnar nerve. In the interests of patient safety, the results of this assessment were disclosed to the attending anesthesiologist immediately prior to extubation. However, the choice of whether or not to reverse the patient pharmacologically and when to extubate the patient was left to the attending anesthesiologist.

After tracheal extubation, children were transferred to the PACU, where adverse events (postoperative nausea and vomiting, severe pain, severe coughing, stridor/obstruction, broncho- or laryngospasm, and oxygen desaturation) were recorded by the attending nurses.

### 2.1. Sample Size and Statistical Analysis

The sampling period was not determined by a formal sample size calculation but simply by the desire to capture data over the equivalent of 1 calendar month [12].

Subsequently, all data were analyzed using IBM SPSS Statistics Version 19 (IBM; Armonk, NY; USA). To compare the incidence of RNMB between different groups of patients, the Chi-square test was used. ANOVA was used to compare data between patients with or without TOF ratios of <0.9. *P* was set at 0.05. Data are displayed as mean (SD).

## 3. Results

During the 5-week study period, a total of 94 tracheally intubated children were screened for inclusion in this audit. Of those 94 children, 30 children were excluded as no NMBA was subsequently given in these cases.

Data of 64 children (8.2 (5.9) yrs; 35.1 (22.6) kg) were analyzed.

Patient characteristics and NMBA details are provided in Table 1.

The administration of an additional intraoperative dose of NMBA was given following a request for prolonged neuromuscular paralysis by the surgeon in all cases.

Neuromuscular monitoring was not routinely used (total *n* = 15 (23.4%); TOF *n* = 14; PTC *n* = 1), such that, in 49 children, the attending anesthesiologist used no neuromuscular monitoring at all.

Reversal was deemed to be required (by attending anesthesiologist) in 33 (51.6%) children. In this instance, neostigmine was given in 32 cases and sugammadex in 1 patient. Neostigmine was always dosed as 0.08 mg kg^−1^ and combined with atropine 0.02 mg kg^−1^.

The incidence of RNMB (TOFr < 0.9) was overall 28.1% (without reversal: 19.4%; after neostigmine: 37.5%; *P* = 0.164). Severe RNMB (TOF ratio < 0.7) was found in 2 patients (6.5%) after both no reversal and neostigmine, respectively. A significant (*P* < 0.001) positive correlation (*r* = 0.33) was found between the time from last NMBA administration and the TOF ratio prior to tracheal extubation.

In 31 (48.4%) cases, reversal was deemed unnecessary by the attending anesthesiologist. However, at the time of independent TOF assessment immediately prior to tracheal extubation, 7/31 of those children had a TOF ratio <0.9 with 2 having a TOF ratio <0.7 (individual TOF ratios directly prior to extubation 34, 65, 75, 83, 85, 88, and 89, resp.). This occurred after disclosure of the TOF ratio assessments to the anesthesiologists. None of the anesthesiologists elected to change their decision to extubate based on these TOF ratios and no reversal agents were administered in these patients.

There was a small but significant difference in the time from skin closure to tracheal extubation between the patients who received no reversal (13.2 (5.5) min) versus the ones who received neostigmine (16.7 (11.4) min; *P* = 0.0134). Incidents in PACU were low, with no differences between the patients receiving reversal versus no reversal (Table 2). Postoperative nausea was found in 6.9% of patients with a TOF ratio above 0.9 versus 15.4% below 0.9 (*P* = 0.576). The incidence of vomiting in PACU was not significantly different between the groups (TOF > 0.9: 10.7% versus TOF < 0.9: 23.1%; *P* = 0.361).

## 4. Discussion

Our prospective audit in 64 children confirmed that RNMB still is a frequently encountered problem at the end of surgery. The overall rate of RNMB (28.1%) found in our study is certainly in line with those reported in adult patients [8] and slightly higher than the latest report about RNMB in children [9].

However, since there is no doubt about the link of RNMB to significant postoperative morbidity in both adults and children [2, 8, 9], any incidence of RNMB may be of concern. Of specific concern is the high incidence of RNMB after reversal of a neuromuscular block with neostigmine (37.5%). Although this incidence seems high in our audit, similar results have been previously reported [7, 12]. In a very large retrospective data analysis involving almost 36000 patients receiving intermediate acting NMBA, Grosse-Sundrup et al. found neostigmine to be an independent risk factor for postoperative oxygen desaturation below 90% and subsequent reintubation [7].

The main contributors to the increased risk of respiratory complications in the PACU were related to the inability to fully reverse deeper levels of neuromuscular blockade, as well as the long and variable onset times of neostigmine. Anesthesiologists may underestimate the onset time and reversal effects in regard to the administration of neostigmine, particularly since the use of neuromuscular monitoring is not routine [2].

Often, anesthetists do not wait a sufficiently long time following the administration of neostigmine for onset of effect. The significant but very small (mean 3.5 min) difference in the time observed from the time of surgical skin closure to the time of tracheal extubation found between patients with no versus neostigmine-based NMBA reversal may reflect the fact that anesthesiologist did not wait sufficiently long enough after the administration of neostigmine.

In our study, only a fraction (23%) of anesthesiologists made use of intraoperative neuromuscular monitoring after administering NMBA. Similarly low rates of monitoring have been previously published [13]. Of the 31 children in whom the attending anesthesiologists had deemed reversal not to be required, 7 children had a TOF ratio below 0.9 with two having values below 0.7. We were surprised to find that the decision not to administer a reversal agent prior to extubation was made despite the timely disclosure of the TOF results to the attending anesthesiologist. This, again, clearly reflects the lack of appreciation for the well-known complications associated with RNMB. In this respect, it has to be emphasized that the latter does not simply represent the mindset of an isolated group of anesthesiologists but rather is a widespread practice worldwide [8].

This observational trial has several limitations. Although we could show that the disclosure of the TOF ratio prior to tracheal extubation did not affect the practice of the attending anesthesiologist, it may still have created a bias (the practice of anesthesiologists aware of the audit may have differed from their usual behavior). Furthermore, we excluded very small children, emergency procedures, and children having surgery outside normal working hours. Though auditing for the equivalent of one calendar month is thought to yield representative results, the number of children in whom NMBA were used (our study population, *n* = 64) might have ultimately been too low to achieve statistical significance, with no significant differences demonstrated between the two groups (i.e., rate of RNMB in children with no reversal versus neostigmine).

We conclude that the incidence of RNMB at the time of tracheal extubation in children remains high. The well-known problems associated with nonreversibility of a deep block with neostigmine as well as the unreliable onset time and effectiveness of the drug are contributors to the specifically high incidence of RNMB after reversal with neostigmine. The lack of utilization of routine neuromuscular monitoring and poor understanding regarding the consequences of RNMB demonstrate a need for better education to increase greater awareness regarding this issue.

## Figures and Tables

**Table 1:** Patient characteristics (ASA: American Society of Anesthesiology, NMBA: neuromuscular blocking agent); total *n* = 64.

ASA category (*n*)	I: 34
	II: 20
	III: 10

Procedure (*n*)	General: 24
	Orthopedic: 7
	Urology: 11
	ENT: 5
	Plastics: 4
	Eye: 2
	Other: 11

Urgency (*n*)	Elective: 44
	Urgent: 20

NMBA on induction (*n*)	Atracurium: 41
	Rocuronium: 15
	Succinylcholine: 6
	Vecuronium: 1

NMBA during maintenance (*n*)	Atracurium: 10
	Rocuronium: 2
	Vecuronium: 1
	Mivacurium: 1

**Table 2:** Incidence (%) of adverse events in the recovery room in patients with (train-of-four [TOF] ratio <0.9) versus without (train-of-four [TOF] ratio >0.9) residual neuromuscular blockade. All differences were found to be not significant.

	TOFr < 0.9	TOFr > 0.9
Bronchospasm (*n* [%])	0	0
Laryngospasm (*n* [%])	0	2 (3.2)
Coughing (*n* [%])	1 (5.3)	2 (3.2)
Oxygen desaturation less than 95% (*n* [%])	2 (10.5)	8 (12.9)
Airway obstruction (*n* [%])	0	2 (3.2)
